# Presidential Address

**Published:** 1915-03

**Authors:** John V. Woodruff

**Affiliations:** Buffalo


### Presidential Address.

DR. JOHN V. WOODRUFF, Buffalo.

During the year 1914 the Erie County Medical Society has achieved some results that will be remembered in its history. We can congratulate ourselves upon having accomplished all that was fondly hoped for by the most optomistic in the way of recognition of Erie County by the State society. Not only was the State Presidency secured, but several of our members have been honored in a manner befitting the numerical strength of the second county society in the State of New York, and the next meeting of the State Society will take place in Buffalo. For these signal recognitions we have to thank particularly several members of the "Old Guard" who have long been on the firing line and whose modesty so nearly equals their political acumen that I hardly dare mention their names for fear of offending them. We must also bear in mind that we owe not a little to the unbounded courtesy and good fellowship shown our delegates by the delegates and members of the county societies at the Eastern end of the State and also to the loyal support of the Western counties. The general popularity and excellent record of our worthy brother who was the recipient of the honor bestowed upon him in being elevated to the State Presidency, greatly aided the efforts of the untiring workers in his behalf, whose sagacious plans won the day. We firmly believe that the successful termination of the efforts of our delegates presage much good to Erie County and the State Society as well. It is perhaps providential that just at this time a hard headed, close observer, forceful and untiring worker like Dr. Wende happened to be placed at the helm, for these are times when the very best and most efficient man should guide the medical barque through what promises to be a stormy passage, especially for the near future. Smooth sailing has in many ways departed and there are indications of breakers ahead where the waters strike the shores of Legislation, Workingmen's Compensation Fee Bill and Fraternal Society. Taking a general view of the field of medicine one cannot but observe omens of marked changes in many ways. The great developments wrought by Bacteriological and Biological discoveries bid fair to be equalled by correspondingly as great changes on the economic side of the field, and the Medical Society of the County of Erie has not failed to see the great change in conditions coming and has endeavored to prepare for it by establishing a Department of Economics the birth of which department in the year 1914 will undoubtedly markedly influence the destinies of the profession in Erie County.

The greatest and most beloved of American Statesmen,

Abraham Lincoln, once declared that the interest of the whole people could not be held upon more than one subject at one time and in keeping with this dictum from so high a source I want to invite your attention this evening to the one subject of Economics as it applies to the practice of medicine and the effect reflected upon the profession from two standpoints, viz.: Contract work and Legislation.

In a general way, the present European War, destined to cause more destruction than anything that has hitherto happened in the history of the world, illustrates the importance of Economics in all human enterprises and endeavors, constituting as it does a veritable octopus, with its innumerable tentacles ramifying through the warp and woof of the entire fabric, entering into the remotest recesses and constituting a cohesive or disruptive force among the elementary parts of the body politic in accordance with the proper or improper application of its laws.

The subject of Economics, from the standpoint of Medical Contract Work has been one of the conspicuous and much mooted topics before this Society for some years back. It has occasioned the expression of much difference of opinion and some feeling of antagonism on the part of some of our members on both sides of the arena and I am sorry to say that up to the present time nothing definite has been accomplished in the way of substantially ameliorating the condition that confronts us, which fact is lamented by practically the whole membership of our society. This condition, if conclusions may be drawn from expressions of an overwhelming majority of our members, would be abolished, even by those personally interested in contract practice, if the precise manner in which contract society work, and other unfortunate practices, bearing directly upon the economics of medical and surgical work, could be worked out for its abolition. The enormous growth of contract work in the past few years render it imperative that measures be adopted for its abolition or control. It is estimated that over two thirds of the male population of Buffalo in adult life are entitled to medical treatment through the payment of one dollar per individual and lately whole families are being taken into this method of obtaining cheap medical aid for the sum of two dollars per year per family. An attempt is being made to extend this sort of practice to the country towns. If this is achieved it will be almost an impossiblity to abolish or control such a condition. A knowledge of these matters on the part of those interested, fortunately, has culminated in the establishment by this society of the before mentioned department of economics, represented in the Council of the society, thus constituting a permanent commission to examine into this intricate subject in all its aspects and make recommends for the consideration of the members of the society. It is hoped that through this means justice may ultimately be done the members of the Medical Profession, financially, in increasing the infinitesimally small fees for visits, and morally in increasing the respect of the members for each other and a higher estimate of the profession by the public. Lastly, and by no means of least importance, it is hoped that justice may be meted out to our patients through their receiving more proper and painstaking medical treatment, which many of us believe they are not getting, as a rule, under the existing order of things as exemplified by contract society practice. This means of disposing of this important problem seems the best and most feasible manner of solving it effectually and it is hoped that in this way the much needed reforms will be satisfactorily arranged for.

Before departing from this subject I feel that I cannot too strongly urge the earnest consideration and co-operation of all members of the Erie County Medical Society concerning this vital problem. It is one that must be satisfactorily solved if the medical profession retains the respect and confidence of the public. And bear in mind it is the status of the commoner here, as elsewhere, that gives immediate strength and vitality. The general practitioner must be protected. If there is continued procrastination we shall most assuredly suffer from our lack of forethought and determination. The surgeon has hitherto considered himself exempt, to a very large extent, from the evils of contract work and analogous methods of dispensing medical aid for a mere pittance in cities, but let me call your attention to the important fact that the Workingman's Compensation Fee Bill is a very potent means of putting the surgeon on the same footing with the general practitioner. Improved technique and better medical education, and especially the greater opportunity of acquiring surgical mechanics, have resulted in very largely increasing the numbers of those capable of doing good surgery, thereby increasing the supply of those competing in this line of work. It is plain, therefore, that surgical prostitution is just as much to be feared as medical slavery. If developments in surgical lines proceed as they have in medical lines in cities the mechanics of brick laying will be ahead of those of surgery, the odds being in favor of the bricklayer for the reason that he has less worry and less responsibility and will have just as much remuneration when expenses are considered. This exemplifies the benefit of co-operation among workers. It seems incomprehensible that co-operation among federated mechanics and laborers is so much easier of attainment than among medical men. Can we admit that greater intelligence leads to disruption and lack of cohesion and harmony? Or is there something lacking in the education of the physician that unfits him to co-operate harmoniously with his brother practitioner? If so we had better appeal to the college to have the matter remedied in order that the Hippocratic oath and the unwritten Hippocratic law once again regain ascendency.

In an address delivered before the Fourteenth Annual Conference of Sanitary Officers, Saratoga Springs, on September 15, 1914, by Governor Glynn, he concluded his paper in the following words:

"You have something to do for yourselves. I do not want to scold or chide you, but when I think how many physicians there are in this state, more than 15,000 of them, with all their influence, and knowing the value of maintaining the medical standards of this State, and remember that you are sleeping dormant while cults are forcing these bills through the Legislature, I feel that you almost deserve that such measures should become laws. These bills should not have been allowed to get to the executive chamber. It was not fair to the Governor to allow them to come and it was not fair to yourselves to allow them to come. I suppose that you have a legislative committee. Well, if you have, make it bigger, busier and better and protect yourselves."

These unequivocal words coming from the executive of the state explain themselves, and the fact that they speak the truth in a very plain, straight forward manner is only too apparent to all of us. It seems to be the general concensus of opinion that the medical profession, as a whole, is asleep, insofar as taking measures to protect themselves from bad legislation at Albany is concerned. We have, so far as I can discern, never had the proper facilities for carrying on a campaign against such legislative measures as those vetoed by Governor Glynn, which as he says, very properly, never should have arrived at the executive chamber. So far as I can learn the course pursued by our legislative committee, has been to go to Albany and protest against the passing of certain bills after they had progressed so far that they are very apt to become laws from the Governor's signature. The Governor insinuates that they should have been nipped in the bud, and I am informed by those who claim to be posted in these matters that the easiest way of disposing of such things is to stop them in their infancy. I do not think that our Legislative Committees have been to blame in this matter. It goes without saying that they cannot spend all their time at Albany watching and waiting to see if something inimical to the medical profession comes up. They are consequently not apprised of such occurrences until such a time as a fairly advanced condition of the bills pending gives them a notoriety and by that time so much work has been done and money spent in various ways that those back of them strain every nerve to put them through, and it is therefore, a much greater task to nullify them. It seems to be the opinion of many who are in position to know the ropes, that the Medical Society of the State of New York should have a paid representative (or representatives) at Albany, whose business it should be to watch for the appearance of measures touching upon the practice of medicine and inform the Legislative Committees of the gist of the matters in hand in order that they may duly consider the trend of such proposed laws and advise the Albany representatives what course to pursue. This is a day of multiplicity of laws and the use of great artfulness in getting them through legislative bodies. It behooves all who can in any way be influenced by legislative matters to band together and protect their interests by keeping a close watch upon Albany. Reading between the lines of the Governor's message to the medical profession it is not difficult to draw an inference as to his full meaning. As a matter of fact it takes one of the initiated in the art of lobbying to offset the arts and wiles of the legislative cult employed by those who are continually threatening the public with bad legislation. It is notoriously a fact that the easiest way of disposing of these matters is in the employment of tact and diplomacy while they are still in committee. It is far easier to pigeonhole and lay them on the table than to evolve elaborate arguments for their non-acceptance by the governor as laws, and the sooner we learn to appreciate the advice of the gentleman who vetoed some dangerous measures in our interests the sooner we will be placed in a position where such obnoxious measures will not menace ourselves and the public.

I understand that this matter of having a paid representative at Albany has been up before the Council of the State Society and rejected on two grounds, viz.: insufficient funds and a sacrifice of the dignity of the profession to conduct our defence in this manner.

So far as the insufficiency of funds is concerned it would seem that every physician in the State would be willing to contribute a dollar or two towards so worthy a cause if appealed to in the proper manner.

Respecting our unwillingness to enter politics I would say that we possess some of the most astute politicians extent and they are not at all slow in using their political acumen in furthering their individual ends. In fact a certain amount of political sagacity is necessary in all legitimate enterprises, be they what they may, and the greater and more far reaching the enterprises the greater the need for tact and diplomacy. Worthiness and ability constitute only a portion of the necessary acquirements for success. Furthermore the greatest men the world has produced have not by any means disdained to further their projects by political aid. Lincoln was a most skillful politician and to his political skill and judgement we owe the fact that we are still a united nation. Franklin exhibited his greatness as a politician in France when he played one faction against another so sagaciously that he finally prevailed in his endeavor to give John Paul Jones the Bonhomme Richard to win a victory with. Jefferson, Hamilton and Hancock were all politicians in the right way and there is no reason why a physician should not delve in politics sufficiently to protect himself and the public. The medical profession is regarded as unselfish and untiring in its efforts to guard the public safety and it hardly seems as though this prestige will be lost through wholesome efforts in restricting dangerous legislation.

I have used my prerogative as retiring officer of this society to bring these homely and common place matters to your notice because it seemed imperative that due notice be given them. A pyramid cannot be built upon its apex and it is just as true that you cannot expect science to rest upon a poor, beggarly class of medical men. Fanancial prosperity is necessary in the interests of all projects. Even Billy Sunday finds that a bowl of soup enables him to create more enthusiasm in his evangelical efforts than a bowl of water. It is the prosperous condition of the great middle class that gives strength and stability, according to Goldsmith, and we must not neglect the general practitioner. The attractive effulgence of the shooting stars in the medical firmament do not lend immediate strength. It is not the man who demands $10,000.00 from the Hills and the Astors that makes up the bone and sinew of the medical host. It is the man who is glad to get $100.00 for an operation or for attendance that we must protect and upon whom devolves the chief work of holding the public respect and support. What other class of men who consider themselves entitled to be called a profession can we find that is willing to allow others to make out their bills and enact laws governing their practice? The managers of contract society work and of legislative enactments at Albany practically do these two things for the physicians. The education of the physician is such that he is peculiarly susceptible to the machinations of those who have any sort of business proposition to exploit. If, as Spencer defines life, it is an adjustment of internal conditions to external surroundings, it seems necessary to consider our internal conditions very closely and endeavor to readjust them on a more rational basis of business judgement.

To recapitulate, the interests of the physician demands that contract practice be abolished, or be arranged upon an entirely different basis, and that attempts at pernicious legislation be watched and circumvented.

In conclusion I wish to bring to your attention some lines much admired by Emerson that seem very apropos to our present condition.

> "Man is his own star,
> And the soul that can render an honest,
> And a perfect man,
> Command all light, all influence, all fate,
> Nothing to him falls too early or too late.
> Our acts our angels are, or good or ill,
> Our fatal shadows that walk by us still.

---

**Relation of Movable Kidney to Trauma.** Theodor Bratrud, Warren, Minn., Railway Surg. Jour., Dec., 1914. Various authorities are quoted, for and against the possibility of dislocation of the kidney by trauma. Peak and others report circumrenal haemorrhage (We are grateful that there is one man who says circum—and peri—) which, with resulting decapsulation is a plausible method of action of trauma in loosening a kidney from its supports. Many surgeons deny the possibility of traumatic loosening of a kidney. Peak reports one case in which a kidney was found loose after an injury and had not been so before. (Note. We have encountered a number of cases in which third degree movability of the kidney apparently followed injury. In some of these, there was positive evidence that the kidney had previously been in this condition; in others there was no record of previous examination. We recall a case in which a surgeon did a nephropexy for movability which we personally knew did not exist. Later the case was referred back to us "because the kidney had become loose again"—but it was just as firm as it had been before and after the nephropexy. Without denying the possibility of traumatic cause of movable kidney, we can say with certainty that we have never been able to produce a single case as evidence. Even such a report as Peak's is not positive evidence for it is well known that the condition of the kidney cannot always be determined at a single examination or even several, without anaesthesia).

---

**Cystin Calculi.** Neumann, Deutsche Med. Woch., Dec. 10, 1914, states that 170 cases have been reported altogether. He has had two cases. In one, a girl of 24, cystinuria attracted attention. X-ray showed calculus in right renal pelvis, left kidney apparently normal. Operation successful but doubt as to further progress of case. Rest in bed, milk diet, sodium bicarbonate embodied the treatment. Just how the X-ray examination revealed the calculus is not stated.

